# Military and Naval Hospitals

**Published:** 1893-12-09

**Authors:** 


### The Institutional Workshop.

### HOSPITALS OF THE WORLD.*

### VI.—MILITARY AND NAVAL HOSPITALS.

The original plan of hospital arrangement for our army was on the regimental system, each battalion having its own hospital, medicine chest, &c.; but in 1793 the Army Medical Board, composed chiefly of men whose experience had covered merely London hospital work, was formed, and a new system, based on civilian lines, was introduced. Several large general hospitals were built for the troops, of which the one at Chatham is the only one used for its original purpose. The new arrangements were not found practicable, and the Army Board was replaced, in 1814, by the creation of a Director-General of the Medical Department. This office was held for thirty-eight years by Sir James McGrigor, principal medical officer under the Duke of Wellington in the Peninsular War. During his administration the regimental system was restored; the amount of hospital accommodation was calculated at five per cent. of the strength in ordinary times. The medical staff for each regiment was one surgeon and two assistants. The attendance on the sick and the cooking were carried on by men taken from the ranks, who received no special training except what they could pick up in the hospital. The hospital buildings were of the most varied description, commonly adapted hastily for hospital purposes, and ill-suited for the accommodation of the sick. The ventilation was very imperfect, and overcrowding was common.

In 1857 a Royal Commission was appointed to inquire into the sanitary condition of the army and the state of the hospitals, and from that period a new order of things was gradually introduced. The rules of construction now generally followed in military hospitals are known as the pavilion plan, and were drawn up under the direction of Sir Douglas Galton and the late Dr. Sutherland. The Herbert Hospital at Woolwich was the first built under this plan, and is still one of the best examples of a pavilion hospital in this country.

The classification of military hospitals embraces three main classes, general, lunatic, and station hospitals. The general hospitals are organised under a principal medical officer, a sanitary officer, and a registrar. In addition to the staff of medical officers, there is usually a lady superintendent and a complement of nursing sisters. These general hospitals may be for (*a*) the local sick of the regular forces; (*b*) foreign invalids (*i.e.*, troops invalided from foreign service); (*c*) seamen and marines of the Royal Navy, foreign sailors, and others admitted under special sanction; (*d*) sick of auxiliary forces; (*e*) sick officers; (*f*) sick women and children. The lunatic hospitals are merely intended for observation and are not permanent asylums. The station hospitals are for the reception of the sick from all corps in the garrison in the command.

Hut hospitals are constructed as occasion requires. They consist of wooden huts, with or without verandahs, according to climate. Six hundred cubic feet of space per head, about half of what is allowed in permanent buildings, is laid down as the minimum in temperate climates. Each ward is a separate hut, the kitchens and offices being also entirely separate. These hut hospitals have been found to be very satisfactory for the treatment of the sick. Special precautions are necessary to regulate the temperature.

In time of war an entirely separate organisation is established. The medical officers attached to each battalion of infantry and regiment of cavalry render assistance on the field of battle itself. Next in order come the bearer companies of the Medical Staff Corps for carrying the wounded to the field hospitals. Each company consists of three medical officers, one warrant officer, and sixty non-commissioned officers and men. Each division has three field hospitals, forming the second line of assistance. These field hospitals are equipped for 100 patients. Stationary hospitals are formed on the lines of communication, and form the third line of assistance. They are, of course, arranged for movement in any case of emergency, and are equipped for 200 beds. The regulation number of these stationary hospitals for an army corps is eight, but the number varies according to the actual requirements. Finally, at the base of operations a general hospital is organised, limited to 500 beds, under the care of the nursing sisters and 118 non-commissioned officers and men, in addition to the medical staff. If no buildings are available, all these hospitals may be constructed of tents. Those used are of two kinds—marquees containing eighteen beds, and circular tents to hold four. In times of emergency the numbers are increased. Great care is taken to keep up a constant movement of the wounded towards the base of operations, in order to ensure sufficient space for the newly wounded near the scene of action. The system is supplemented by the use of hospital ships, which are found excellent for the purpose. Each ship is equipped for 200 or 250 patients. When stationary as a depôt, each hospital ship has attached to it one or more fast steam vessels, each making up 60 beds, to act as relieving ships to remove the worst cases to England or elsewhere. A small steam transport is also attached to each depôt ship to provide for the commissariat. The organisation is completed by the provision of small despatch vessels for the transference of slighter cases to steam packets on their way to England. On the whole, the English soldier wounded in battle in some distant part of the world stands as good a chance of skilled attention as if he were knocked down by a hansom cab in a London street; and, undoubtedly, a far better chance of careful transport.

In America the establishment of military hospitals dates from the Civil War, when 202 hospitals, with accommodation for 136,594 patients, were constructed. Most of these hospitals were adapted from buildings used for the most varied purposes, such as churches, hotels, factories, and private dwellings. Gradually these were superseded by the building of wooden pavilions. Most of those now in use are of this description. Many are merely temporary. The War Department places the entire control of the military hospitals under the medical officers. They are mostly small buildings, containing from twelve to forty-eight beds each. The supply of furniture, instruments, books, and hospital stores is a very liberal one, and the money voted by Congress for this purpose is under the control of the Surgeon-General of the Army.

In the German army the organisation of the Medical Department is very thorough. The medical officers are trained either at the Universities or at the Friedrich Wilhelm's Institute at Berlin for aiding and training medical students who are intended for commissions in the Sanitäts Corps. The medical officers have no jurisdiction over the soldiers, even when under treatment in the hospital. In the garrison or general hospitals the nursing is performed by Sisters of Mercy, or other trained nurses. But in time of war female nurses are not employed in the field hospitals; the nurses are men who have served three years' compulsory service. After they have drilled one year with their regiments they are sent for training to the hospital, where they serve on probation for six months.

In France regimental hospitals are established with each battalion, intended only for slight cases. Station hospitals

* "Hospitals and Asylums of the World," 4 vols., and Portfolio of Plans. (Messrs. Churchill and the Scientific Press.)

are established in various towns, but of late years many of these military hospitals have been amalgamated with the civil hospitals. In some, wards are reserved for military patients, who are treated under military regulations. In others, where the garrison is below 300 men, the soldiers are treated in the ordinary wards by civil surgeons. The entire management of military hospitals is under the Army Medical Department. Nursing sisters are employed under the orders of the medical officers. The Systême Tollet, which mainly consists in constructing the roof in the form of an arch, is the one commonly adopted for the military hospitals.

In the Italian army the principal variation is the establishment of regimental infirmaries for the reception of soldiers suffering from slight ailments and needing only simple treatment. They consist of thermal and sea-bathing establishments, and are supplemented by convalescent depôts for receiving patients who have recovered from severe illness in military hospitals.

